# Risk assessment and intervention strategies in controlling HAIS risk in the HCU at Premier Bintaro Hospital

**DOI:** 10.1017/ash.2025.119

**Published:** 2025-09-03

**Authors:** Rosdelima Simarmata, Hesti Maria Banu, Wuri Susilowati, Rumah Sakit Premier Bintaro

**Keywords:** HAIs, Screening, Scoring, HCU

## Abstract

**Introduction:** Hundreds of millions of patients worldwide are infected with Healthcare-Associated Infections (HAIs) each year causing deaths and significant financial losses to health systems. HAIs incidence in Indonesia (2021) reached 15.74%, far above in developed countries (4.8-15.5%). At Premier Bintaro Hospital (RSPB), the number of High Care Unit (HCU) patients at risk of HAIs reached 51% in the January - June 2023 period. The aim of this research is to reduce the risk of HAIs in HCU patients at RSPB. **Methods:** The HAIs risk identification was conducted on all HCU patients when first admitted using the SIRS (Systemic Inflammatory Response Syndrome) and REDS (Risk Emergency Department Scoring) criteria. Based on the screening results, interventions were applied through environmental modifications, implementation of HAIs care bundle, and nurse allocation based on colour codes: At risk of infection as RED, and no risk of infection as GREEN. The risk monitoring was conducted using HAIs scoring. **Results:** The research was conducted in the January - June 2023 period on 206 patients. It was found that 51% of the patients were at risk of HAIs. After interventions, the number reduced to 39%. In addition, there were zero incidents of HAIs during that period. **Conclusions:** To control the risk of HAIs in HCUs, the implementation of risk screening & scoring and interventions such as environmental modifications, nurse allocation, and HAIs care bundle, have shown to be effective strategies. This is evident from the decrease in the number of patients at risk of HAIs.

Recommendations for hospital is to continue implementing risk control of HAIs by regularly assessing the risk, applying interventions, and consistently monitoring the risk as efforts in continuously reducing the HAIs risks. For future research, it is recommended to expand the sample of patients and extend the period to implement risk control of HAIs even further.

**Acknowledgements:** We are very grateful to all members of CCU team at Premier Bintaro Hospital for their contribution in implementing the methods and strategies of HAIs risk control in this study.

